# Endoscopic Ultrasound-Guided Fine-Needle Aspiration (EUS-FNA) with Image Enhancement

**DOI:** 10.3390/diagnostics10110888

**Published:** 2020-10-30

**Authors:** Masahiro Itonaga, Reiko Ashida, Masayuki Kitano

**Affiliations:** Second Department of Internal Medicine, Wakayama Medical University, 811-1 Kimiidera, Wakayama City, Wakayama 641-0012, Japan; masaitonaga0907@gmail.com (M.I.); rashida@wakayama-med.ac.jp (R.A.)

**Keywords:** EUS-FNA, CH-EUS, EUS-elastography

## Abstract

Although endoscopic ultrasound-guided fine-needle aspiration (EUS-FNA) is useful in the differential diagnosis of solid pancreatic lesions, lymph nodes, and liver lesions, inadequate sampling may result in an incorrect pathological diagnosis. The accuracy of EUS for the evaluation of pancreatobiliary lesions may be increased by image enhancement technologies, including contrast-enhanced harmonic (CH)-EUS and EUS-elastography. These methods can provide information that complement EUS-FNA for the diagnosis and staging of pancreatobiliary cancer, and can help to identify the EUS-FNA target, reducing the requirement for repeat FNA.

## 1. Introduction

Endoscopic ultrasound-guided fine-needle aspiration (EUS-FNA) has been generally used since 1992 for the sampling of pancreatobiliary tissues [1]. However, EUS-FNA is subject to sampling error, which can make some EUS-FNA biopsy specimens inadequate for pathological diagnosis. Due to the possibility of false-negative and nondiagnostic results, selecting an appropriate treatment strategy is challenging for patients with inconclusive EUS-FNA findings [2]. Several device characteristics and methods, such as needle sizes and forms, suction, slow-pull, and fanning techniques, as well as rapid on-site cytological evaluation (ROSE), have been developed to improve the diagnostic performance of EUS-FNA [3,4,5,6]. When sampling errors occur despite the implementation of these technical improvements, EUS-FNA is often repeated, and meta-analysis of the results of repeat EUS-FNA has revealed a sensitivity of 77% and a specificity of 98% [7]. Nevertheless, sampling errors can still occur and in real clinical situations, the final diagnosis is often an image-based diagnosis rather than a pathological diagnosis. In such cases, endosonographers may experience difficulties in identifying the appropriate target of EUS-FNA. Appropriate identification of actual target lesions is important for further improving the diagnostic yield of EUS-FNA.

The accuracy of EUS for the evaluation of pancreatic lesions, lymph nodes, and liver lesions can be increased by image enhancement technologies, including contrast-enhanced harmonic (CH)-EUS and EUS-elastography. At present, both of these methods play important roles in the clinical evaluation of pancreatic cancer [8,9,10,11,12]. CE-EUS and EUS-elastography can provide information complementary to that of EUS-FNA for the diagnosis and staging of pancreatobiliary cancer, and can help to identify the EUS-FNA target, reducing the requirement for repeated EUS-FNA. This review provides an overview of the ability of EUS-FNA with image enhancement to diagnose pancreatic tumors, lymph nodes, and liver tumors.

## 2. Diagnostic Techniques

### 2.1. EUS-FNA

EUS-FNA is currently regarded as the most effective method of acquiring tissue samples from pancreatic lesions. During EUS-FNA, the target lesion is visualized under real-time ultrasound guidance with a linear echoendoscope, blood vessels are excluded by color Doppler, and the lesion is punctured with a needle and sampled by a series of in-and-out needle passes directed in multiple directions within the target lesion, a method called the fanning technique. Samples obtained by EUS-FNA may contain free cells, cell groups, and macroscopically visible tissue fragments, together with blood clots. Samples are processed for cytology or histology. 

The following techniques have been used to increase the accuracy of EUS-FNA: (1) fanning; (2) suction; (3) wet suction; (4) slow pull, (5) ROSE, and (6) EUS-guided fine-needle biopsy.

(1) Fanning

In randomized controlled trials (RCTs) of patients with pancreatic masses, the fanning method resulted in fewer needle passes being required for diagnosis of malignancy than the standard method [6]. Furthermore, the fanning method yielded significantly higher first-pass diagnoses than the standard FNA method (85.7% vs. 57.7%, *p* = 0.02) [6].

(2) Suction

Three RCTs have assessed the impact of negative pressure, performed using a needle attached to a 10- or 20-mL suction syringe, on EUS-FNA results [13,14,15]. These studies predominantly used 22G FNA needles. In two RCTs of patients with pancreatic masses, 10-mL suction improved the sensitivity and accuracy of malignancy compared to no suction. One of these studies also showed that 20-mL suction was superior to 10-mL suction. [13]. By contrast, in RCTs of patients with mediastinal or abdominal lymphadenopathy, suction did not affect the probability of obtaining a correct diagnosis [15].

(3) Wet suction

The “wet suction” technique was developed to enhance tissue acquisition by applying principles of fluid dynamics to the suction technique [16]. The wet technique involves flushing the needle with saline to replace the column of liquid and air, which is less compressible than a gas and better transmits the negative pressure applied to the proximal port of the needle to the tip of the needle [16,17]. In an RCT comparing standard and wet suction for FNA in patients with various solid masses and Lymph Nodes (LNs), the wet suction method improved the adequacy and quality of the samples. However, the impact on diagnostic accuracy was not assessed [16].

(4) Slow pull

The “slow pull” technique is performed by progressive withdrawal of the stylet as the needle repeatedly passes through the target lesion. This produces a low level of negative pressure in the needle that increases tissue acquisition and reduces blood in the specimen. In a retrospective study of 97 cases of EUS-FNA that were performed with either the slow pull technique or conventional suction, the slow pull technique reduced cellularity and blood contamination when a 25-gauge FNA needle was used, but not when a 22-gauge needle was used, resulting in higher diagnostic sensitivity [18]. The current meta-analysis reveals that the slow pull technique was not superior to conventional suction, although it resulted in reduced blood contamination [19].

(5) Rapid on-site cytological evaluation (ROSE)

ROSE has been proposed as a strategy to improve diagnostic yield. ROSE may improve the efficiency of the procedure and reduce patient risk by limiting the number of needle passes. The presence of an on-site cytopathologist has been reported to improve diagnostic rates by 10–15% [20]. An inadequate sample rate of about 20% has been reported when ROSE is not adopted [21]. On the other hand, ROSE is not logistically feasible and may increase procedural costs. For solid pancreatic masses, Erickson et al. [22] reported that well-differentiated pancreatic adenocarcinomas required a higher number of passes (5.5 ± 2.7) than moderately (2.7 ± 1.2) and poorly (2.3 ± 1.1) differentiated tumors. Pellisé Urquiza et al. [23] reported that the accuracy of the EUS-FNA for pancreatic masses reached a plateau after the fourth needle pass. Turner et al. [24] reported in a large cohort of 559 patients with pancreatic masses that a diagnostic accuracy of approximately 80% could be obtained after only two to three needle passes. Similarly, Wallace MB et al. [15] reported that lymph nodes generally require only two to three passes. Therefore, in the absence of ROSE, four to five passes are recommended for solid pancreatic lesions and two to three passes for lymph node, liver, and adrenal lesions [25].

(6) EUS-guided fine-needle biopsy (EUS-FNB)

Traditionally, only a EUS-FNA needle has been available for tissue sampling. The early EUS-FNB needle was a true-cut biopsy needle, but it was difficult to puncture some targets with this type of needle because of poor penetration. Currently, EUS-FNB needles are available in a variety of sizes ranging from 19 gauge to 25 gauge and include reverse bevel, fork-tip, and Franseen needles. These needles are very easy to use, as are EUS-FNA needles. Four recent meta-analyses comparing EUS-FNA with EUS-FNB for the analysis of solid pancreatic lesions [26,27,28,29] revealed that fewer needle passes were required for EUS-FNB, although there was no significant difference in the diagnostic yield between EUS-FNA and EUS-FNB when EUS-FNA was performed by ROSE. On the other hand, in the absence of ROSE, EUS-FNB had a relatively good diagnostic accuracy, suggesting that EUS-FNB is preferable when ROSE is not available. A meta-analysis revealed a significant difference in diagnostic yield between EUS-FNA and EUS-FNB in subepithelial lesions, but not in pancreatic lesions [30].

### 2.2. CH-EUS

CH-EUS, which was developed in 2008 [31], involves the administration through a peripheral vein of contrast agents, consisting of gas-filled microbubbles approximately 2–5 µm in diameter, encapsulated by a phospholipid or lipid shell [32]. Transmitted US waves disrupt or stimulate these microbubbles to resonate, thereby producing the harmonic signals detected in the US image, with few artifacts. CH-EUS enables the selective observation of signals from the microbubbles (contrast agent) by elimination signals from the tissues. 

CH-EUS is more advantageous than contrast-enhanced MRI and CT in patients with contraindications for using intravenous contrast agents, such as renal failure or contrast allergy, although adverse reactions to contrast agents are rare. Another advantage of CH-EUS is that it allows real-time dynamic imaging and repeated examinations.

The CH-EUS procedure involves the following steps: The mechanical index (MI) is set to 0.2–0.4. A bolus injection of contrast agent (Sonazoid, 0.015 mL/kg body; Sonovue, 2.4–4.8 mL/body; Definity, 10 mL/kg body) is administered through a peripheral vein. Before infusion of the contrast agent, the following four procedures must be implemented.

1. The echo image of CH-EUS should be lowered to a level at which almost no tissue signal is detected.
2. The focus point is set to the lowest point of the lesion.
3. The fundamental B-mode image is simultaneously depicted as a monitor image in order to facilitate orientation during contrast enhancement.
4. Because attenuation makes it more difficult to observe deep lesions than Fundamental B mode, the area where the lesions are observed in close proximity to each other should be searched before contrast enhancement.

In the normal pancreas, signals from the contrast agent appear between 10 and 15 s, and peak at approximately 20 s, after administration of the contrast agent. CH-EUS can be analyzed quantitatively using inflow time mapping and time-intensity curve (TIC) patterns to characterize the lesions.

### 2.3. EUS-Elastography

Elastography equipment can be coupled with conventional EUS without the need for an additional agent. Two types of EUS-elastography, strain and shear wave, have been developed. Strain elastography estimates the stiffness of the target tissue by measuring the degree of strain produced in response to compression. Shear wave elastography involves the emission of focused US from the probe to the target tissue, called the acoustic radiation force impulse (ARFI). The stiffness of the target tissue can then be estimated by measuring the propagation speed of the shear wave.

When performing EUS elastography, the pancreatobiliary region is first screened by EUS, and then, the targeted region of interest (ROI) is narrowed down. Next, the scope is held at a site where the object can be observed in detail in B mode. In addition, the object is rotated so that it appears in the 6 o’clock position on the screen during radial EUS. While remaining aware of aortic and other arterial pulsations in abdomen, the probe is then held lightly against the gastrointestinal wall to prevent vibration. A good elastography image is one that is stable over a period of time (usually more than 5 s) and that achieves uniform resolution throughout the ROI. A strain graph displayed on the screen is used as a reference to determine whether an appropriate image has been obtained and to evaluate the stability of the image. The strain graph shows the compression intensity and distortion, and when the graph shows maximum elongation under compression caused by beating of the aorta, the image is judged to have been rendered with the desired vibration energy. Evaluation of images near the top of the waveform of the graph is preferable. However, if the distance from the aorta is too great, proper images may not be obtained in cases of severe atherosclerosis. If it is not possible to obtain an effective vibration, a position change must be performed at the site where the lesion is sandwiched between the probe and the aorta. 

## 3. Solid Pancreatic Lesions

### 3.1. Diagnostic Yields of EUS-FNA

Four meta-analyses have reported that the sensitivities and specificities of EUS-FNA for the diagnosis of pancreatic cancer were 85–92% and 96–98%, respectively [33,34,35,36]. The tissue acquisition and safety of EUS-FNA have been shown to be superior to those of other methods, such as endoscopic retrograde cholangiopancreatography (ERCP). A study of 8246 patients who underwent EUS-FNA reported that the overall rate of complications was 0.82%, with rates of pain, bleeding, and pancreatitis being 0.38%, 0.10%, and 0.4%, respectively [37].

### 3.2. Complementary Role of CH-EUS Relative to EUS-FNA

CH-EUS categorizes solid lesions in the pancreas into three to four patterns based on their intensity of enhancement: non-enhancement, hypo-enhancement, iso-enhancement, and hyper-enhancement. CH-EUS images of pancreatic cancers are hypo-enhanced, with a lower intensity of enhancement than surrounding pancreatic tissue. In the most extensive series to date, consisting of 277 patients, most pancreatic solid lesions exhibited a pattern of hypo-enhancement on CH-EUS [38]. Hypo-enhancement on CH-EUS in patients with Sonazoid-diagnosed pancreatic cancer had a sensitivity of 95% and a specificity of 89%. In that report, 36 (78%) of 46 inflammatory masses showed iso-enhancement, whereas 15 (78%) of 19 neuroendocrine tumors (NETs) showed hyper-enhancement [39]. In three meta-analyses, the pooled sensitivity and specificity of CE-EUS for the diagnosis of pancreatic cancer were 93–94% and 80–89%, respectively [8,39,40]. 

Three studies compared CH-EUS with EUS-FNA for the diagnosis of pancreatic cancer [38,41,42]. In these, hypo-enhancement on CH-EUS had a sensitivity of 89–96% and a specificity of 88–94% for diagnosing pancreatic cancer, comparable to EUS-FNA, which had a sensitivity of 72–95% and a specificity of 100%. However, 80–100% of lesions with false-negative diagnoses by EUS-FNA were correctly diagnosed by CH-EUS. These results suggest that CH-EUS could help to decide between surgery and observation when the results of EUS-FNA are inconclusive.

### 3.3. Targeting EUS-FNA with CH-EUS 

**Figure 1:**
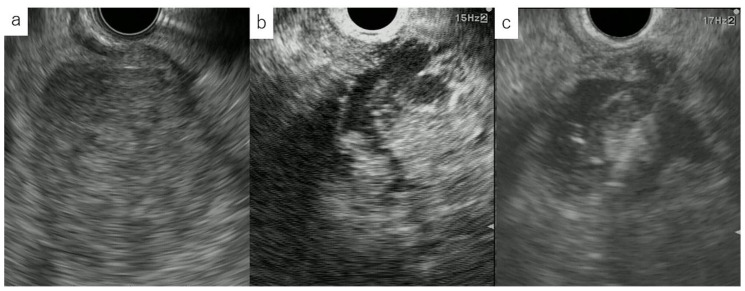
Targeting EUS-FNA with CH-EUS. (**a**) Fundamental B mode endoscopic ultrasonogram showing a low echoic mass. (**b**) CH-EUS image showing a non-enhancing area within the tumor. (**c**) EUS-FNA with CH-EUS avoiding the non-enhancing area.

CH-EUS enables better visualization of fibrotic and necrotic areas in pancreatic lesions, areas that may be overlooked in biopsy specimens. Avascular areas in pancreatic adenocarcinomas have been associated with severe fibrosis and necrosis within these lesions [43]. In addition, avascular (non-enhancing) areas in pancreatic lesions on CH-EUS have been reported to represent areas of necrosis, with EUS-FNA having sensitivities of 72.9% (35/48) and 94.3% (230/244) for lesions with and without avascular areas, respectively, sensitivities that differed significantly [44]. Moreover, samples obtained by puncturing avascular (non-enhancing) areas on CH-EUS were found to contain necrotic tissue and to therefore be inadequate for histological diagnosis, whereas samples obtained by puncturing vascular areas contained malignant cells [45]. By avoiding non-enhanced areas on CH-EUS, the combination of EUS-FNA and CH-EUS would likely have better diagnostic ability than conventional EUS-FNA (Figure 1). Five studies examined the ability of EUS-FNA with or without CH-EUS to target solid pancreatic lesions [46,47,48,49,50] (Table 1).One study reported that the percentage of adequate biopsy specimens obtained by EUS-FNA with CH-EUS (96.6%) was higher than that obtained by conventional EUS-FNA (86.7%) [46]. Another study found that EUS-FNA with CH-EUS required fewer needle passes than conventional EUS-FNA to obtain adequate samples from solid pancreatic lesions [47]. However, all five studies found that EUS-FNA with CH-EUS did not improve the sensitivity and accuracy of conventional EUS-FNA for malignancy in patients with pancreatic masses.

EUS-FNA with CH-EUS has been found to improve the visualization of pancreatic lesions compared with conventional EUS [51]. Fundamental B mode EUS sometimes fails to visualize pancreatic tumors, especially in patients with chronic pancreatitis, diffusely infiltrating carcinomas, or a recent episode of acute pancreatitis [52]. EUS-FNA with CH-EUS can improve the visualization of pancreatic lesions and the identification of different pathological areas within these lesions. While heterogeneous areas on CH-EUS are indicative of a tumor, homogeneous areas are associated with inflammation of the tissues surrounding the tumor. The rates of adequate sampling and sensitivity were found to be significantly higher with EUS-FNA with CH-EUS than with conventional EUS-FNA, as the former avoids non-enhancing and homogeneous areas in hypo-enhancing lesions [53] (Table 1). Thus, CH-EUS improves the ability of EUS-FNA to identify pancreatic lesions and targets areas within these lesions for sampling.

**Table 1:** Targeting endoscopic ultrasound-guided fine-needle aspiration (EUS-FNA) with contrast-enhanced harmonic (CH)-EUS for pancreatic solid lesions.

First Author, Year	Design Number of Patients	Techniques Compared		Targeting Method on CH-EUS	Outcome Measures	
					Diagnostic Accuracy	Sample Adequacy and Quality
Hou, 2015 [46]	Retrospective, 163 patients	EUS-FNA with CH-EUS (22G)	Conventional EUS-FNA (22G)	Avoiding non-enhancing areas	NSD in sensitivity (81.6% vs. 70.8%) and specificity (100% vs. 100%)	NSD in adequate sampling rate (96.6% vs. 86.7%)
Sugimoto, 2015 [47]	Parallel-group, RCT 40 patients	EUS-FNA with CH-EUS (22G)	Conventional EUS-FNA (22G)	Avoiding non-enhancing areas	NSD in sensitivity (90% vs. 85%) and accuracy (90% vs. 85%) for malignancy	NSD in adequate sampling rate (100% vs. 100%) Higher adequacy rate for the first needle pass in the CH group (60% vs. 25%; *p* = 0.027)
Seicean, 2017 [48]	Prospective, 51 patients	EUS-FNA with CH-EUS (22G)	Conventional EUS-FNA (22G)	Avoiding non-enhancing areas	NSD in sensitivity (82.9% vs. 73.2%) and specificity (100% vs. 100%)	
Seicean, 2020 [49]	Parallel-group, RCT 225 patients	EUS-FNA with CH-EUS (22G)	Conventional EUS-FNA (22G)	Avoiding non-enhancing areas	NSD in sensitivity (87.6% vs. 85.5%) and specificity (100% vs. 100%)	
Facciorusso A 2020 [50]	Retrospective, Propensity Score Matching 206 patients	EUS-FNA with CH-EUS (22G)	Conventional EUS-FNA (22G)	Targeting hypo-enhancing area	NSD in sensitivity (87.6% vs. 80.0%) and specificity (100% vs. 100%)	
Itonaga, 2020 [53]	Prospective, 93 patients	EUS-FNA with CH-EUS (22G)	Conventional EUS-FNA (22G)	Avoiding non-enhancing and homogeneous areas	EUS-FNA with CH-EUS had increased sensitivity (84.9% vs. 68.8%, *p* = 0.003) but NSD in specificity (100% vs. 100%)	EUS-FNA with CH-EUS increased adequate sampling rate (81.2% vs. 67.3%, *p* = 0.003)

NSD: no significant difference.

### 3.4. Complementary Role of EUS-Elastography Relative to EUS-FNA

Strain elastography uses qualitative and quantitative methods. The qualitative evaluation method for strain elastography is color pattern diagnosis, with blue indicating hard tissues and red indicating soft tissues within tumors and patterns that include heterogeneous and homogenous color tones. Elastography patterns can be classified into five types, based on elasticity scores, color patterns, and heterogenicity of distribution of elasticity [54]. EUS-elastography was found to have a sensitivity of 100% and a specificity of 67% in the diagnosis of malignant lesions.

Quantitative evaluation methods have been classified as strain ratio and histogram analysis. The strain ratio is defined as the ratio of the strain on the target lesion to the strain on the peripheral tissue [55]. Histogram analysis involves the evaluation a grayscale histogram, which is created by converting an elastography image into a grayscale image with 256 levels [56]. On EUS-elastography, strain indicating the stiffness of target lesions may help to differentiate harder pancreatic cancers from surrounding tissues. In eight meta-analyses, the pooled sensitivity and specificity of EUS-elastography were 95–99% and 63–76%, respectively [9,57,58,59,60,61,62,63].

Two studies compared EUS-elastography with EUS-FNA for the diagnosis of pancreatic cancers [64,65]. EUS-elastography had a sensitivity of 86–96% and a specificity of 43–66%, whereas EUS-FNA had a sensitivity of 82–90% and a specificity of 100%. However, the combination of EUS-elastography and EUS-FNA showed a high negative predictive value to exclude malignant pancreatic lesions [65]. Moreover, a study in patients with pancreatic masses and negative cytopathology on EUS-FNA found that the combination of CH-EUS and EUS-elastography had a sensitivity of 88.9% and a specificity of 100% [66]. Thus, EUS-elastography may complement EUS-FNA as a diagnostic tool. 

### 3.5. Targeting EUS-FNA with EUS-Elastography

**Figure 2:**
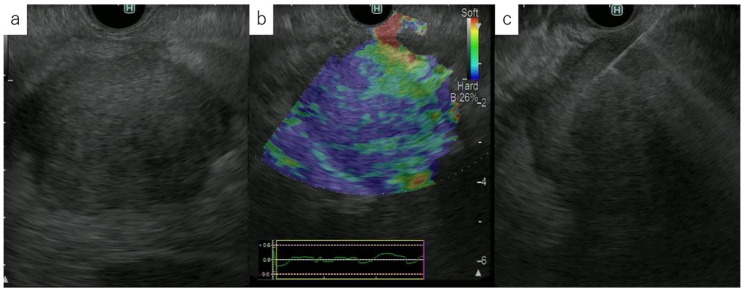
Targeting EUS-FNA with EUS-elastography. (**a**) Fundamental B mode endoscopic ultrasonogram showing a low echoic mass. (**b**) EUS-elastography image showing no distortion on low echo area and surrounding tissue. (**c**) EUS-FNA with EUS-elastography targeting the harder area.

EUS-elastography exploits the difference in tissue elasticity between malignant and benign lesions, and between different parts within the same solid masses, to correctly and precisely target the harder part of the lesion, which is more likely to be malignant. By targeting the harder area, EUS-FNA with EUS-elastography can improve the diagnostic ability of conventional EUS-FNA (Figure 2). Two studies reported that EUS-elastography was an efficient and safe technique for diagnosing solid pancreatic lesions [67,68] (Table 2). However, those studies included relatively few patients and did not include a control cohort of patients undergoing conventional EUS-FNA. Prospective randomized trials are necessary to confirm the usefulness of EUS-FNA with EUS-elastography. 

## 4. Lymph Nodes 

### 4.1. Diagnostic Yields of EUS-FNA

The diagnostic examination of celiac lymph nodes (LNs) is necessary in determining surgical indications for pancreatic cancer. EUS-FNA evaluation of para-aortic LNs was shown to have a sensitivity of 96.7% and a specificity of 100% for the diagnosis of metastases [69]. Moreover, a large meta-analysis reported that EUS-FNA had a sensitivity of 88.0% and a specificity of 96.4% for the diagnosis of malignant LNs [70].

### 4.2. Complementary Role of CH-EUS Relative to EUS-FNA 

A meta-analysis reported that CH-EUS had a sensitivity of 82% and a specificity of 90.7% for the diagnosis of malignant LNs [10].

A comparison of CH-EUS with EUS-FNA showed that they had similar accuracy for the diagnosis of malignant LNs (88% vs. 90%). CH-EUS correctly N-staged three patients who failed EUS-FNA sampling due to LN location [71]. This study reported that lymph nodes are malignant if heterogeneous enhancement is observed on CH-EUS, suggesting that CH-EUS can be used to complement EUS-FNA for the diagnosis of malignant LNs.

### 4.3. Targeting EUS-FNA with CH-EUS

There are no reports to date of CH-EUS targeting EUS-FNA for the diagnosis of malignant LN.

### 4.4. Complementary Role of EUS-Elastography Relative to EUS-FNA 

A meta-analysis reported that EUS-elastography had a sensitivity of 88.0% and a specificity of 85% for the diagnosis of malignant LN [72]. There are no reports describing the complementary roles of EUS-elastography and EUS-FNA for the diagnosis of malignant LNs.

### 4.5. Targeting EUS-FNA with EUS-Elastography

To our knowledge, no study to date has described the use of EUS-elastography targeting EUS-FNA for the diagnosis of malignant LNs.

## 5. Liver Lesions

### 5.1. Diagnostic Yields of EUS-FNA

Before the widespread use of EUS, most liver metastases were diagnosed by either abdominal imaging or percutaneous biopsy. However, EUS can identify very small (<1 cm) liver metastases as well as allowing for concurrent FNA to aid in the staging of certain malignancies. EUS-FNA has a sensitivity of 82–98% for the diagnosis of malignant liver metastases [73,74,75,76,77,78,79].

### 5.2. Complementary Role of CH-EUS Relative to EUS-FNA 

Phagocytosis of Sonazoid by Kupffer cells after the arterial and portal phases results in its distribution into normal liver tissue, generating US images called Kupffer-phase images [80]. Because intact Kupffer cells are absent from metastatic liver tumors, liver metastases in patients with pancreatic cancer are likely to be detected as perfusion defects in Kupffer-phase images obtained by CE-EUS. A study of 426 patients with pancreatic cancer found that CH-EUS had a sensitivity of 96.6% and a specificity of 99.0% in the detection of left liver metastases [81]. By comparison, CE-CT had a sensitivity of 69.8% and a specificity of 98.4%, and conventional EUS had a sensitivity of 76.7% and a specificity of 99.7% [81]. Moreover, CH-EUS, CE-CT, and conventional EUS had sensitivities of 88.9%, 11.1%, and 30.6%, respectively, for the detection of small (<10 mm) liver metastases. To our knowledge, however, no reports to date have described the complementary roles of CH-EUS and EUS-FNA for the diagnosis of liver tumors.

### 5.3. Targeting EUS-FNA with CH-EUS

A study assessing the usefulness of CH-EUS for EUS-FNA in the detection of hepatic lesions found that, before contrast enhancement, 73.3% of the hepatic lesions were visible on B mode [82]. After contrast enhancement, however, 93.3% of these hepatic lesions were distinguishable from the surrounding liver parenchyma. Thus, EUS-FNA with CH-EUS had a technical success rate of 100% and a diagnostic accuracy of 86.7%. 

### 5.4. Complementary Role of EUS-Elastography Relative to EUS-FNA 

The ability of EUS-elastography to distinguish between benign and malignant conditions is based on the greater hardness of malignant diseases compared with benign lesions and normal conditions. For example, EUS-elastography was reported to have a sensitivity of 92.5% and a specificity of 88.8% for the diagnosis of malignant liver lesions [83]. To our knowledge, however, no reports to date have described the complementary roles of EUS-elastography and EUS-FNA in the diagnosis of liver tumors.

### 5.5. Targeting EUS-FNA with EUS-Elastography

To date, no studies have described the use of EUS-elastography to target EUS-FNA in the diagnosis of liver tumors.

## 6. Conclusions

EUS-FNA has high sensitivity and specificity for the diagnosis of pancreatobiliary lesions. Because of possible sampling errors, however, some biopsy specimens obtained by EUS-FNA are inadequate for appropriate pathological diagnosis. Methods of enhancing EUS images, such as CH-EUS and EUS-elastography, can provide complementary information on the diagnosis and staging of pancreatobiliary lesions. Although the diagnostic yields of EUS-FNA are generally good, these yields can be improved by methods that recognize appropriate areas in the tumor for sampling.

## Figures and Tables

**Table 2:** Targeting EUS-FNA with EUS-elastography for pancreatic solid lesions.

First Author	Design, Number of Patients			Diagnostic Accuracy	Sample Adequacy and Quality
Jafri, 2016 [67]	Retrospective Single Arm, 8 patients	EUS-FNA with EUS-elastography (22G or 25G)	target the “harder” area	Sensitivity (100%) and specificity (100%)	
Facciorusso A 2018 [68]	Retrospective Single Arm, 54 patients	EUS-FNA with EUS-elastography (25G)	target the “harder” area	Sensitivity (93.4%) and specificity (100%)	Adequate sampling rate (98.2%)
